# Trends in added sugars intake and sources among U.S. adults using the National Health and Nutrition Examination Survey (NHANES) 2001–2018

**DOI:** 10.3389/fnut.2022.897952

**Published:** 2022-08-18

**Authors:** Loretta DiFrancesco, Victor L. Fulgoni, P. Courtney Gaine, Maria O. Scott, Laurie Ricciuto

**Affiliations:** ^1^Source! Nutrition, Toronto, ON, Canada; ^2^Nutrition Impact, LLC, Battle Creek, MI, United States; ^3^The Sugar Association, Inc., Washington, DC, United States; ^4^Department of Nutritional Sciences, University of Toronto, Toronto, ON, Canada

**Keywords:** added sugars, trends, intake, sources, U.S., adults, NHANES

## Abstract

Research on trends over time in added sugars intake is important to help gain insights into how population intakes change with evolving dietary guidelines and policies on reducing added sugars. The purpose of this study was to provide an analysis of dietary trends in added sugars intakes and sources among U.S. adults from 2001 to 2018, with a focus on variations according to the sociodemographic factors, age, sex, race and ethnicity and income, and the health-related factors, physical activity and body weight. Data from nine consecutive 2 year cycles of the National Health and Nutrition Examination Survey (NHANES) were combined and regression analyses were conducted to test for trends in added sugars intake and sources from 2001 to 2018. Trends were examined in the whole sample (19+ years) and in subsamples stratified by age (19–50, 51+ years), sex, race and ethnicity (Asian, Black, Hispanic, White), household income (poverty income ratio low, medium, high), physical activity level (sedentary, moderate, vigorous) and body weight status (normal, overweight, obese). From 2001 to 2018, added sugars intake (% kcal) decreased significantly (*P* < 0.01), from 16.2 to 12.7% among younger adults (19–50 years), mainly due to declines in added sugars from sweetened beverages, which remained the top source. There were no changes in intake among older adults, and by 2018, the 23% difference in intake between younger and older adults that existed in 2001 almost disappeared. Declines in added sugars intake were similar among Black and White individuals, and all income, physical activity and body weight groups. Population-wide reductions in added sugars intake among younger adults over an 18 year time span coincide with the increasing public health focus on reducing added sugars intake. With the updated Nutrition Facts label now displaying added sugars content, it remains to be seen how added sugars intake trends carry forward in the future.

## Introduction

Over the past two decades, population guidelines on dietary sugars intake have evolved, culminating in 2015 with international guidelines from WHO to reduce intake of “free sugars” (including added sugars and those naturally present in honey, syrups, fruit juices and fruit concentrates) to <10% of calories per day (1). In the U.S., recommendations on added sugars intake are included in the Dietary Guidelines for Americans (DGA), first appearing in 1980 with a general statement about avoiding too much sugar (2), and then developing to a more specific recommendation to reduce intake to <10% of calories per day, initiated in 2015 (2) and maintained in the 2020 DGA (3). With the evolution of the DGA to a quantitative recommendation on added sugars intake, there have been product reformulations to reduce added sugars content in some foods and beverages (4). In a number of cities, taxes have also been imposed on sugar-sweetened beverages to discourage their consumption (5), and even prior to 2015, public health messaging focused on reducing sugar-sweetened beverage consumption. Research on trends in added sugars intake and the food sources contributing to these trends has thus become increasingly important to help gain insights into how population intakes might have changed over time in this context of evolving guidelines and public heath focus on reducing added sugars intake.

Examinations of trends in added sugars intake in the U.S. have been conducted over different time spans and among different age groups, and taken together they provide evidence of significant declines in intake among all age groups over the years 1999–2018 (6–12), largely driven by reductions in added sugars from sweetened beverages (13, 14). Yet despite these declining trends in added sugars, intakes remain above 10% of calories and therefore warrant continued monitoring and examination. Furthermore, updates to the Nutrition Facts label were phased in from 2016 to 2021, requiring the declaration of added sugars content and percent Daily Value on food labels (15), and so trends in intake before this time period can provide a baseline against which potential shifts in the consumption of various sources of added sugars may occur.

There are also documented variations in added sugars intake according to the sociodemographic factors, age, race and ethnicity and income (4, 6–8, 11, 12, 16–19). Highest added sugars intakes have been observed among teens and younger adults (12, 16, 17), and Black individuals and low income groups also tend to have the highest added sugars intakes (11, 12, 20). Asian individuals tend to have the lowest intakes (20), and White individuals and high income groups tend to show the greatest reductions over time in added sugars intake (7, 8). A rigorous examination of added sugars intake would therefore include analyses among various population subgroups in order to reveal any disparities in trends, which would be particularly relevant in the milieu of population-level interventions.

Additionally, trends in added sugars intake may vary according to health-related factors, such as physical activity and body weight status, but the limited research on the association between these factors and added sugars intake has shown inconsistent results (21–24); therefore, an examination of added sugars intake trends according to physical activity and body weight status would contribute to the limited knowledge in this area.

The purpose of this study was to conduct a comprehensive analysis of trends over time in added sugars intake and sources among U.S. adults (19+ years) from 2001 to 2018, with a focus on variations according to the sociodemographic factors, age, sex, race and ethnicity and income, and the health-related factors, physical activity level and body weight status.

## Materials and methods

### Data

Nationally representative data from NHANES were used for this analysis. Data from nine consecutive 2 year cycles of NHANES were combined, starting with the 2001–02 cycle and ending with the 2017–18 cycle. The NHANES is a cross-sectional survey of the non-institutionalized civilian U.S. resident population 2+ years; details on the survey design, data collection and analytic procedures are reported elsewhere (25, 26).

### Added sugars intake

Dietary intake data in NHANES are collected from two non-consecutive 24-h recalls using the five-step Automated Multiple Pass Method administered by trained interviewers (26). The USDA Food Patterns and Equivalents Database (FPED) is used to convert food and beverage intakes to food pattern equivalents, corresponding to those in the DGA (27). The added sugars food pattern component is comprised of caloric sweeteners using the definition of added sugars as “sugars that are added to foods as an ingredient during preparation, processing or at the table; and do not include naturally occurring sugars such as lactose present in milk and fructose present in whole or cut fruit and 100% fruit juice” (28).

Mean added sugars intake was determined for each NHANES cycle using the cycle-specific FPED and Day-1 intake data, which is sufficient for providing an accurate estimate of population mean intake (29). Mean added sugars intake was calculated as a percentage of total daily calories (% kcal) in order to account for differences in energy intake over time, and the population ratio method was used for this calculation. The population ratio was selected over the mean ratio method because every individual contributes equally to the overall mean, regardless of their added sugar intake level, and thus provides a better reflection of usual intake at the population level (30). The population ratio method involves summing the daily added sugars intake for all individuals and then dividing by the sum of total daily calories for the same individuals.

In order to further understand added sugars intake trends, trends in food sources of added sugars were also analyzed over the same time period. Sources of added sugars were based on the 2017–18 What We Eat in America (WWEIA) food categories, in which foods and beverages are grouped according to their similar nutrient content and common use in the diet; and individual food categories can be combined into larger food groups for analytical purposes (26) (Table 1). Mean added sugars intake from each food source was calculated as a percentage of total daily added sugars intake using the population ratio method; food sources were then ranked from highest to lowest.

**Table 1:** Breakdown of food groups into types of foods [WWEIA categories (26)] that provide added sugars.

**Food group**	**Food category**
Breads, Rolls, Tortillas	Yeast breads; Rolls and buns; Bagels and English muffins; Tortillas
Candy	Candy: containing chocolate; not containing chocolate
Coffee and Tea	Coffee; Tea
Fats and Oils	Butter and animal fats; Margarine; Cream cheese, sour cream, whipped cream; cream and cream substitutes; Mayonnaise; Salad dressings and vegetable oils
Flavored Milk	Flavored milk: whole; reduced fat; lowfat; non-fat
Other Desserts	Ice cream and frozen dairy desserts; Pudding; Gelatins, ices, sorbets
Quick Breads and Bread Products	Biscuits, muffins, quick breads; Pancakes, waffles, French toast
Ready-to-eat Cereals	RTE cereal: higher sugar (>21.2 g/100 g); lower sugar (<21.2 g/100 g)
Sugars	Sugars and honey; Sugar substitutes; Jams, syrups, toppings
Sweet Bakery Products	Cakes and pies; Cookies and brownies; Doughnuts, sweet rolls, pastries
Sweetened Beverages	Soft drinks; Fruit drinks; Sport and energy drinks; Nutritional beverages; Smoothies and grain drinks
Yogurt	Yogurt: regular; Greek

RTE, ready-to-eat; WWEIA, What We Eat in America.

### Statistical analyses

Data were analyzed using SAS 9.4 (SAS Institute, Cary, NC, USA). Weighting factors provided by NHANES were applied to the combined sample of the nine NHANES cycles, in order to adjust for the complex survey sampling design, sample design changes across survey cycles, non-response rates and oversampling of certain subgroups. Linear and quadratic regression analyses were used to test for trends over time in added sugars intake, with estimated mean added sugars intake as the dependent variable and NHANES cycle as the continuous independent variable. Linear regression analyses were also used to compare mean added sugars intake in each NHANES cycle to the reference cycle, 2001–02. The same analyses were conducted on food sources of added sugars, with those contributing at least 2% to total daily added sugars intake based on NHANES 2001–02 considered for analysis.

In order to assess variations in added sugars trends over time among different population subgroups, analyses were stratified according to several sociodemographic and health-related factors. The full sample of adults (19+ years) was stratified by age (19–50, 51–70, and 71+ years) and sex. Preliminary analyses revealed similar patterns among adults 51–70 and 71+ years, and thus these two age categories were collapsed into one group, representing “older adults.” The sample was also stratified according to the NHANES categorization for race and ethnicity: Hispanic, non-Hispanic Asian (hereafter, Asian), non-Hispanic Black (hereafter, Black), and non-Hispanic White (hereafter, White); household poverty income ratio (PIR) of low, medium and high (PIR < 1.35, 1.35 < PIR < 1.85, and PIR > 1.85, respectively); physical activity level as sedentary, moderate and vigorous, based on the number of days in which vigorous exercise was performed using the NHANES physical activity questionnaire responses (0–3, 4–6, and 7 days per week, respectively); and, body weight status of normal (BMI 18.5–24.9), overweight (BMI 25.0–29.9), and obese (BMI ≥ 30). Trends in added sugars intakes were examined in all strata for each age group, and trends in food sources were examined in all strata only for the overall age group (19+ years) in order to have a large enough sample size. For the Hispanic and Asian ethnic groups, nationally representative data were only available starting with NHANES 2007–08 and 2011–12, respectively; trend analyses for these two groups were thus conducted on the combined sample from 2011 to 2018 to facilitate direct comparisons. A *P*-value of < 0.01 was used to determine statistical significance.

## Results

Using data from the nine cycles of NHANES, representing the time span from 2001 to 2018, the final sample size of all individuals 2+ years was *n* = 72,829, after excluding those with missing or unreliable data (*n* = 10,163), pregnant or lactating females (*n* = 1,631), and kcal = 0 (*n* = 6). The final analytic sample size of adults 19+ years was 44,572, with *n* = 23,552 younger adults (19–50 years) and *n* = 21,020 older adults (51+ years).

### Added sugars trends by age and sex

**Figure 1:**
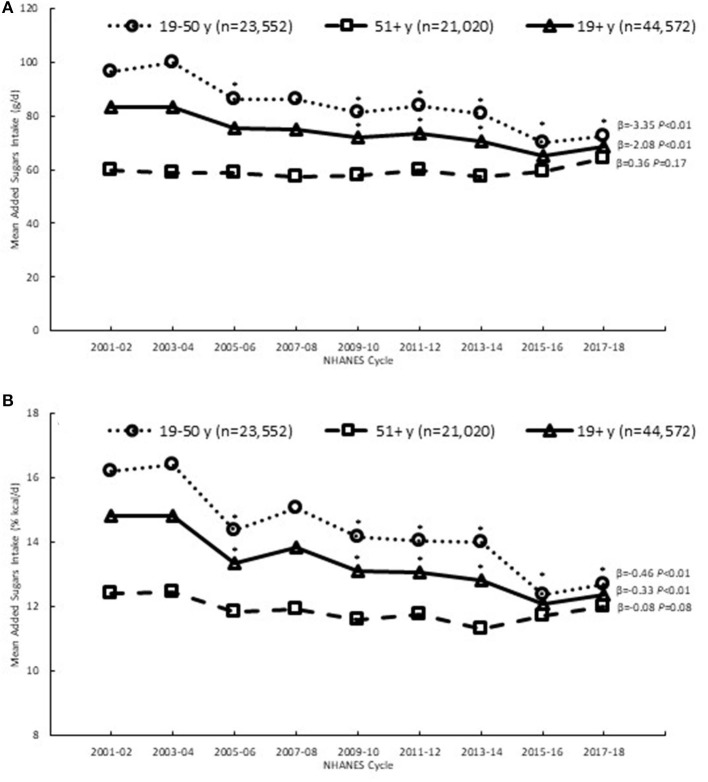
Added sugars intake **(A)** (g/d) and **(B)** (% kcal) among adults, 2001–2018, based on first day of dietary recall; β and *P*-values from linear trend analysis; *Significantly different from reference cycle (NHANES 2001–02) and trend significant at *P* < 0.01; Source NHANES 2001–02 to 2017–18.

Daily added sugars intake (grams and % kcal) decreased significantly over time among adults 19+ years, which was solely due to significant declines among younger adults (19–50 years), as there were no significant declines observed among older adults (51+ years) (Figure 1). Among younger adults, there was an average 3.4 gram decrease in added sugars intake with every cycle, representing an overall decline of 25% from 2001 to 2018 (Supplementary Table 1). For relative intake, added sugars declined from 16.2 to 12.7% kcal among younger adults, representing an overall decline in magnitude of 22% (Supplementary Table 2). At the initial time point, added sugars intake was 23% higher among younger adults compared to older adults, but by 2018, intakes were similar for younger and older adults, with intakes among younger adults 6% higher than those among older adults.

The decreasing trends in added sugars intake from 2001 to 2018 among younger adults could be largely attributed to decreasing consumption of added sugars from sweetened beverages, as their contribution to total daily added sugars intake decreased significantly from 49.7 to 37.7% (Table 2), yet they remained the number one source. A significant decrease in the contribution from other desserts was also observed among younger adults, but to a much smaller extent than the decline in sweetened beverages. In contrast, the contribution from coffee and tea increased significantly over time from 4.8% in NHANES 2001–02 to 10.4% in 2017–18, becoming the second highest source of added sugars. Among older adults, there were no significant trends in the sources of added sugars, except for a significant increase in the added sugars contribution from coffee and tea (Table 3), similar to the trend observed among younger adults.

**Table 2:** Trends in sources^a^ of added sugars among adults (19–50 y, *n* = 23,552), 2001–2018:^b^ food group contributions as a percent of total daily added sugars intake.

**Food group^c^**	**Sweetened Beverages**		**Sweet Bakery Products**		**Sugars**		**Candy**		**Coffee and Tea**		**Other Desserts**		**Ready-to-eat Cereals**		**Breads, Rolls, Tortillas**		**TAS** **All Sources**	
**Year**	**Mean^d^ (SE) % TAS**	***P***	**Mean (SE) % TAS**	***P***	**Mean (SE) % TAS**	***P***	**Mean (SE) % TAS**	***P***	**Mean (SE) % TAS**	***P***	**Mean (SE) % TAS**	***P***	**Mean (SE) % TAS**	***P***	**Mean (SE) % TAS**	***P***	**Mean (SE) g**	***P***
2001–02	49.7 (1.28)		10.4 (0.66)		7.2 (0.43)		6.2 (0.48)		4.8 (0.48)		4.7 (0.39)		2.9 (0.32)		2.1 (0.10)		96.6 (2.74)	
2003–04	52.5 (1.16)	0.10	11.1 (0.76)	0.51	5.9 (0.48)	0.04	5.4 (0.31)	0.13	4.6 (0.66)	0.77	4.1 (0.38)	0.29	2.5 (0.23)	0.25	2.0 (0.12)	0.59	100.1 (2.81)	0.37
2005–06	47.0 (1.21)	0.12	12.2 (0.60)	0.05	6.5 (0.41)	0.24	5.5 (0.64)	0.38	5.4 (0.69)	0.48	5.0 (0.70)	0.66	2.8 (0.18)	0.76	2.0 (0.09)	0.84	86.4 (2.62)	0.00^e^
2007–08	47.3 (2.30)	0.36	10.3 (0.70)	0.86	6.6 (0.25)	0.19	6.0 (0.77)	0.82	6.0 (0.69)	0.15	4.6 (0.31)	0.86	2.8 (0.25)	0.79	2.1 (0.10)	0.95	86.3 (4.05)	0.04
2009–10	45.7 (1.08)	0.02	10.7 (0.54)	0.73	5.8 (0.33)	0.00^e^	5.7 (0.35)	0.36	7.4 (0.78)	0.00^e^	4.5 (0.34)	0.78	3.1 (0.27)	0.71	2.5 (0.08)	0.00^e^	81.5 (1.96)	0.00^e^
2011–12	42.4 (1.47)	0.00^e^	12.0 (0.67)	0.09	6.7 (0.55)	0.47	4.4 (0.30)	0.00^e^	8.9 (0.66)	0.00^e^	4.5 (0.72)	0.79	3.3 (0.27)	0.39	2.1 (0.16)	0.75	83.6 (2.29)	0.00^e^
2013–14	42.9 (1.57)	0.00^e^	11.2 (0.46)	0.34	5.9 (0.39)	0.02	5.8 (0.59)	0.58	8.3 (0.97)	0.00^e^	3.9 (0.27)	0.11	2.5 (0.14)	0.20	2.0 (0.06)	0.76	80.7 (1.96)	0.00^e^
2015–16	40.0 (1.74)	0.00^e^	10.7 (0.76)	0.80	6.2 (0.62)	0.18	5.1 (0.55)	0.11	10.1 (0.97)	0.00^e^	3.5 (0.26)	0.01	3.0 (0.34)	0.84	1.8 (0.11)	0.12	70.0 (2.51)	0.00^e^
2017–18	37.7 (2.15)	0.00^e^	10.3 (0.84)	0.87	6.7 (0.61)	0.48	6.3 (0.74)	0.96	10.4 (1.10)	0.00^e^	3.6 (0.49)	0.07	2.9 (0.20)	0.99	1.5 (0.13)	0.00^e^	72.3 (2.69)	0.00^e^
**Trend**	**Beta (SE)**	***P***	**Beta (SE)**	***P***	**Beta (SE)**	***P***	**Beta (SE)**	***P***	**Beta (SE)**	***P***	**Beta (SE)**	***P***	**Beta (SE)**	***P***	**Beta (SE)**	***P***	**Beta (SE)**	***P***
Linear	−1.61 (0.23)	0.00^f^	−0.02 (0.10)	0.86	−0.08 (0.07)	0.30	−0.09 (0.10)	0.39	0.77 (0.08)	0.00^f^	−0.15 (0.04)	0.00^f^	0.00 (0.04)	0.93	−0.03 (0.03)	0.37	−3.35 (0.35)	0.00^f^

SE, standard error; TAS, total added sugars.

a Contributing at least 2% to TAS in reference cycle, NHANES 2001–02;

b Source NHANES 2001–02 to 2017–18;

c 2017–18 What We Eat in America food groups;

d Based on Day-1 intake data;

e Significantly different (P < 0.01) from reference cycle, NHANES 2001–02;

f Significant (P < 0.01) linear trend.

**Table 3:** Trends in sources^a^ of added sugars among adults (51+ y, *n* = 21,020), 2001–2018:^b^ food group contributions as a percent of total daily added sugars intake.

**Food group^c^**	**Sweetened Beverages**		**Sweet Bakery Products**		**Sugars**		**Other Desserts**		**Candy**		**Coffee and Tea**		**Ready-to-eat Cereals**		**Breads, Rolls, Tortillas**		**TAS All Sources**	
**Year**	**Mean^d^ (SE) % TAS**	***P***	**Mean (SE) % TAS**	***P***	**Mean (SE) % TAS**	***P***	**Mean (SE) % TAS**	***P***	**Mean (SE) % TAS**	***P***	**Mean (SE) % TAS**	***P***	**Mean (SE) % TAS**	***P***	**Mean (SE) % TAS**	***P***	**Mean (SE) g**	***P***
2001–02	31.8 (2.25)		16.4 (0.89)		10.5 (0.58)		8.8 (0.52)		5.8 (0.61)		3.9 (0.60)		3.7 (0.41)		3.5 (0.17)		59.9 (2.28)	
2003–04	30.2 (1.73)	0.59	18.7 (1.42)	0.16	8.2 (0.75)	0.02	7.8 (0.78)	0.30	6.3 (0.51)	0.51	4.4 (0.54)	0.46	3.0 (0.21)	0.18	3.4 (0.14)	0.54	58.7 (1.51)	0.66
2005–06	28.7 (1.14)	0.23	17.3 (1.08)	0.51	9.5 (0.64)	0.26	8.2 (0.53)	0.44	7.4 (1.01)	0.17	4.5 (0.51)	0.38	3.1 (0.32)	0.26	3.7 (0.20)	0.42	58.9 (2.48)	0.78
2007–08	26.0 (1.34)	0.03	18.3 (0.98)	0.16	8.7 (0.56)	0.03	8.5 (0.59)	0.75	7.9 (0.70)	0.02	5.5 (0.69)	0.07	3.6 (0.34)	0.90	3.5 (0.14)	0.74	57.5 (1.63)	0.40
2009–10	24.0 (0.85)	0.00^e^	16.1 (0.55)	0.79	9.2 (0.53)	0.12	9.0 (0.54)	0.82	6.7 (0.64)	0.32	7.9 (1.17)	0.00^e^	3.7 (0.23)	0.10	3.8 (0.21)	0.27	58.0 (1.59)	0.51
2011–12	26.5 (1.34)	0.04	16.3 (0.96)	0.93	9.3 (0.89)	0.25	7.5 (0.77)	0.15	6.8 (0.62)	0.23	7.9 (1.57)	0.02	3.6 (0.30)	0.92	3.3 (0.13)	0.21	59.7 (1.98)	0.97
2013–14	24.2 (1.67)	0.00^e^	18.4 (1.00)	0.14	9.1 (0.53)	0.09	6.9 (0.46)	0.00^e^	7.0 (0.92)	0.25	8.0 (1.09)	0.00^e^	3.6 (0.30)	0.85	3.4 (0.13)	0.60	57.4 (1.72)	0.40
2015–16	23.2 (1.64)	0.00^e^	15.2 (1.06)	0.41	9.3 (0.80)	0.22	6.9 (0.82)	0.06	5.2 (0.59)	0.53	12.3 (1.67)	0.00^e^	3.2 (0.37)	0.38	2.5 (0.13)	0.00^e^	59.2 (1.43)	0.81
2017–18	25.9 (1.93)	0.05	16.0 (0.96)	0.76	8.9 (0.48)	0.03	6.9 (0.65)	0.02	6.5 (0.73)	0.48	8.2 (1.22)	0.00^e^	2.6 (0.17)	0.02	2.1 (0.20)	0.00^e^	64.0 (2.17)	0.19
**Trend**	**Beta (SE)**	***P***	**Beta (SE)**	***P***	**Beta (SE)**	***P***	**Beta (SE)**	***P***	**Beta (SE)**	***P***	**Beta (SE)**	***P***	**Beta (SE)**	***P***	**Beta (SE)**	***P***	**Beta (SE)**	***P***
Linear	−0.89 (0.30)	0.02	−0.14 (0.16)	0.41	−0.09 (0.07)	0.26	−0.25 (0.08)	0.01	−0.03 (0.11)	0.80	0.76 (0.14)	0.00^f^	−0.07 (0.05)	0.24	−0.13 (0.05)	0.03	0.36 (0.26)	0.17

SE, standard error; TAS, total added sugars.

a Contributing at least 2% to TAS in reference cycle, NHANES 2001–02;

b Source NHANES 2001–02 to 2017–18;

c 2017–18 What We Eat in America food groups;

d Based on Day-1 intake data;

e Significantly different (P < 0.01) from reference cycle, NHANES 2001–02;

f Significant (P < 0.01) linear trend.

Trends in added sugars intake over time were similar for males and females, with significant declines in intake observed among younger adult males and females, and no changes observed among older males and females (data not shown).

### Added sugars trends by race and ethnicity and income

**Figure 2:**
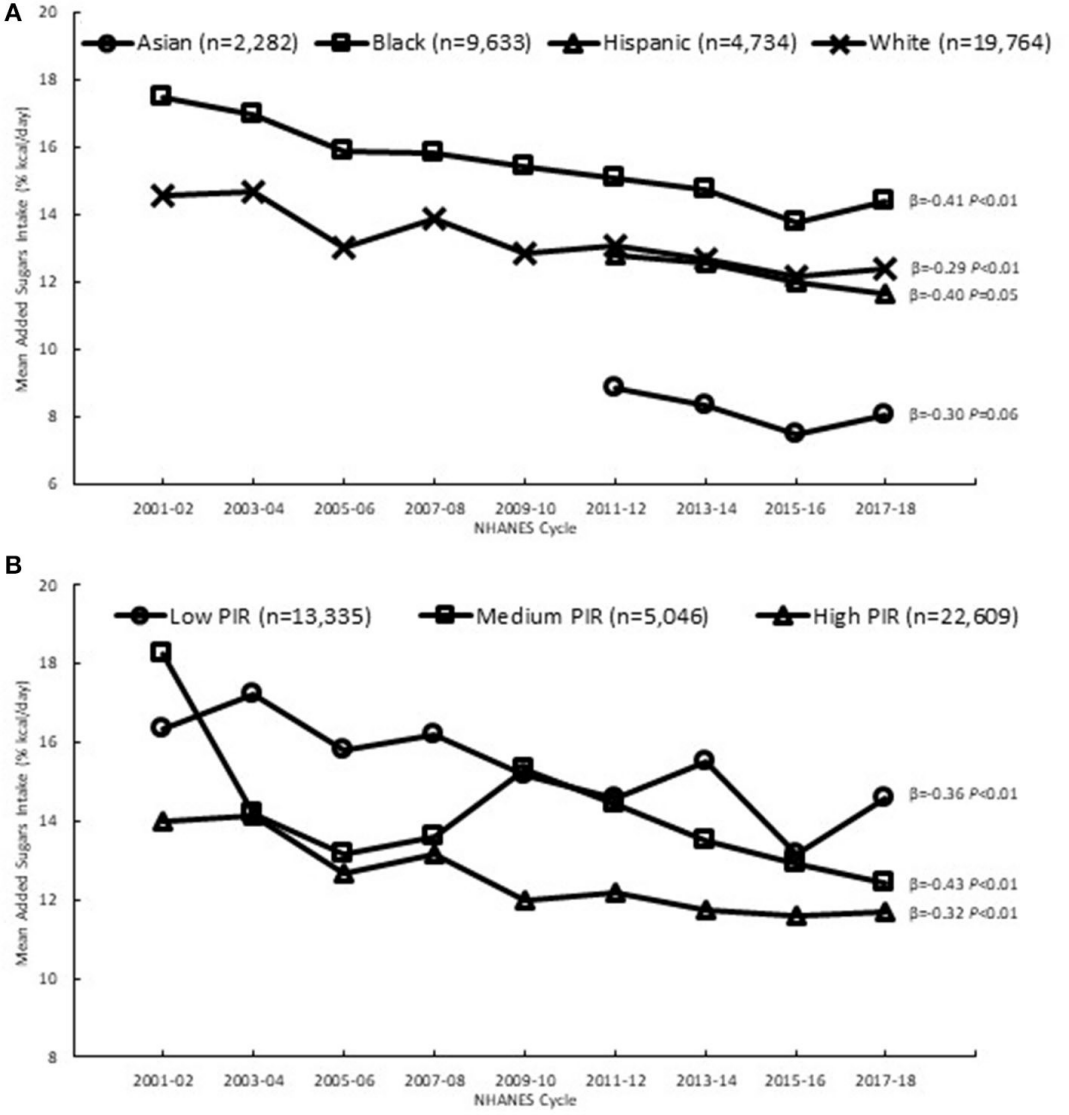
Added sugars intake among adults (19+ y), 2001–2018^*a*^, by **(A)** race and ethnicity and **(B)** income, based on first day of dietary recall; β and *P*-values from linear trend analysis, significant at *P* < 0.01; ^*a*^2011–2018 for Hispanic and Asian individuals to facilitate direct comparisons because nationally representative sample available starting in 2007–08 and 2011–12, respectively; Source NHANES 2001–02 to 2017–18; PIR, poverty income ratio: low (PIR < 1.35), medium (1.35 ≤ PIR ≤ 1.85), and high (PIR > 1.85).

Significant decreasing trends in added sugars intake (% kcal) from 2001 to 2018 were observed among Black individuals in both the younger and older age groups (19–50 years and 51+ years) and among White individuals in the younger age group only (Figure 2A and Supplementary Table 3); the declines in added sugars intake observed among Black and White younger adults were of similar magnitude. There was a downward trend in intake among Hispanic individuals from 2011 to 2018, but this trend did not reach significance, and there was no significant change in intake among Asian individuals from 2011 to 2018. At initial time points, Black individuals had the highest added sugars intake compared to other ethnic groups and Asian individuals had the lowest intakes; this rank order remained the same in 2018.

The decreasing trends in added sugars intake among Black and White individuals could be attributed to significant decreasing trends in the added sugars contribution from sweetened beverages, which remained the top source of added sugars (Supplementary Table 4). The added sugars contribution from coffee and tea increased over time among Black and White individuals, becoming the third highest source of added sugars among both ethnic groups. Among Black individuals only, there was also a significant curvilinear trend in the added sugars contribution from sweet bakery products, whereby their contribution increased at a greater rate in the early years than in the later years, while remaining the second highest source of added sugars.

Significant decreasing trends in added sugars intake (% kcal) from 2001 to 2018 were observed across all PIR groups (Figure 2B and Supplementary Table 3). The magnitude of decline was largest in the medium PIR group, which had the highest added sugars intake initially (18.3%) compared to the low and high PIR groups. These trends could be attributed to significant decreasing trends in the added sugars contribution from sweetened beverages, observed among all PIR groups (Supplementary Table 5). In contrast, there were significant increases over time in the contribution from coffee and tea among all PIR groups.

### Added sugars trends by physical activity level and body weight status

**Figure 3:**
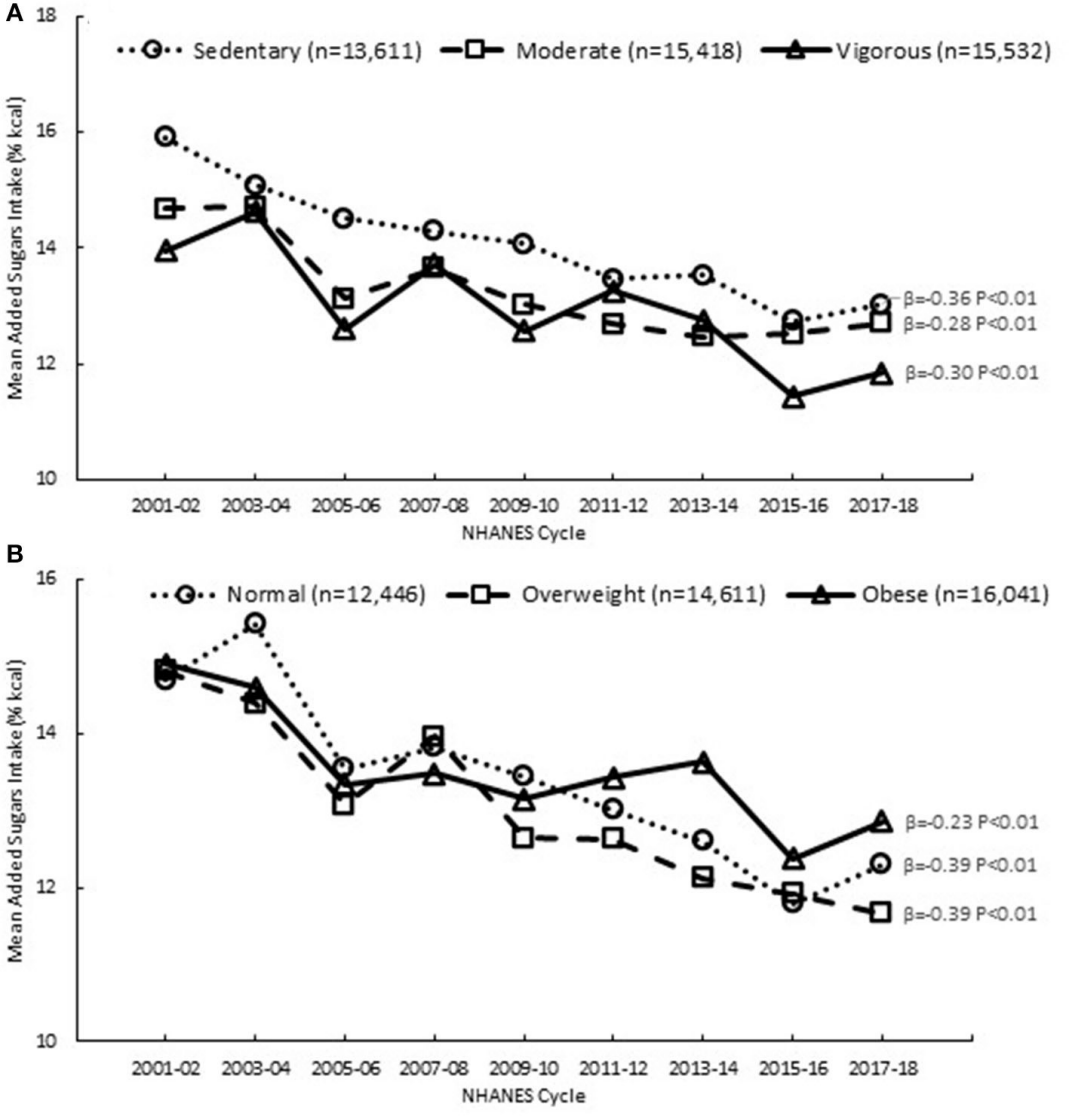
Added sugars intake among adults (19+ y), 2001–2018, by **(A)** physical activity level and **(B)** body weight status, based on first day of dietary recall; β and *P*-values from linear trend analysis, significant at *P* < 0.01; Source NHANES 2001–02 to 2017–18.

Significant declines in added sugars intake were observed among younger adults in all physical activity and body weight groups, with the magnitude of decline varying across the groups (Figure 3 and Supplementary Table 3). The steepest declines were observed among the sedentary and moderate physical activity groups relative to the vigorous activity group, and among the overweight group compared to the normal weight and obese groups. These decreasing trends in added sugars intake could be attributed to significant declines in the added sugars contribution from sweetened beverages among all groups (Supplementary Tables 6, 7). In contrast, there were significant increases over time in the added sugars contribution from other desserts among adults in the vigorous physical activity group; the contribution from coffee and tea also increased significantly in the vigorous activity, normal weight and obese groups.

## Discussion

From 2001 to 2018, added sugars intake declined among younger adults (19–50 years) in the U.S., while intake among older adults (51+ years) did not change. The declining trends in added sugars intake we observed are generally consistent with patterns reported in other studies among U.S. adults, encompassing a similar time span from 1999 to 2018 (7, 8, 10–12), and also align with U.S. food disappearance data showing declines in per capita availability of added sugars from 2001 to 2018 (31). Our results also demonstrate that added sugars intake declined across various sociodemographic groups, defined by race and ethnicity and income, similar to observations in other studies (7, 8, 11, 12, 19, 20). Furthermore, a novel contribution to the literature is our finding that added sugars intake declined among adults in all physical activity levels and body weight groups.

The decline in added sugars intake among younger adults, but not among older adults, could reflect different patterns of dietary intake between these two lifestages (20). Older adults tend to have healthier diets (7, 20) and lower intakes of added sugars (12, 17), and consume fewer sweetened beverages (13, 17, 19) compared to younger adults. With higher intakes of sweetened beverages among younger adults compared to older adults, there might be more room for them to reduce their added sugars intake without requiring substantial shifts in their diet, while this may not be the case for older adults. However, it is also possible that the food and beverage choices of younger adults might be more easily influenced by dietary messaging, and their intakes impacted more by associated interventions compared to older adults. A notable exception to the differences between younger and older adults was the observed increase in added sugars from coffee and tea that was common to both age groups, suggesting a universal appeal of these beverages among adults of all ages. Ultimately, over the 18 year time span, differences in added sugars intake by age became smaller, such that by 2018, added sugars intake (% kcal) were similar among younger and older adults; furthermore, intakes among both groups remained above the recommended DGA reduction to <10% of calories per day.

The decrease in added sugars intake among younger adults (19–50 years) from 2001 to 2018 was due to significant declines in the added sugars contribution from sweetened beverages (soft drinks, fruit drinks, sport and energy drinks, nutritional beverages, smoothies and grain drinks), which remained the top source of added sugars over the 18 year time span even despite the decline. These trends are consistent with other examinations of NHANES data reporting declines in sweetened beverage consumption among younger adults in the U.S. (6, 7, 13, 14, 19), mainly due to reduced consumption of soft drinks and fruit drinks (13, 14). In contrast, the added sugars contribution from coffee and tea increased among both younger and older adults, a trend also observed by other researchers (14). This trend suggests a notable shift in the beverage choices of adults, away from soft drinks and fruit drinks toward sweetened coffee and tea. However, even with the increases in added sugars from coffee and tea, especially among younger adults, sweetened beverages remained the dominant contributor to added sugars intake; ready-to-eat cereals and breads, rolls and tortillas remained minor contributors, together accounting for <5% of added sugar intake.

Several factors could be underlying the decline in added sugars intake among younger adults in the U.S. It might be just one part of an overall trend toward healthier eating, as other aspects of the diet have also improved over time, such as increased consumption of whole grains, fruit and plant-based proteins (6–8). Given the decline we observed was solely due to declines in the added sugars contribution from sweetened beverages, public health policies targeting these beverages likely also contributed (5); and product reformulations and new product launches to reduce the added sugars content in sweetened beverages may have contributed, as suggested by evidence of shifts over time toward the purchase of beverages containing a mixture of both caloric and non-caloric sweeteners (4). Additionally for all foods and beverages, changes to the Nutrition Facts label to include added sugars content and percent Daily Value were phased in from 2016 to 2021 (15), and may have contributed to product changes, but likely to a very small degree, given our sample only went to 2018.

The similar declines in added sugars intake we observed across different race and ethnicities and income levels are generally consistent with patterns documented by other researchers (7, 8, 11, 12, 19). To our knowledge, the similar declines in added sugars intake that we observed across all physical activity levels and body weight status groups have not been documented in other research, as studies on those factors and their relation to added sugars intake appear to be limited to cross-sectional analyses at a single time point (21–24). In the context of population-level interventions to reduce added sugars intakes, our observations of declines in intake in younger adults, that were similar among Black and White individuals and those at all income levels, are encouraging. However, disparities persist, as we observed Black and low income individuals having the highest intakes, vs. Asian and high income individuals groups having the lowest intakes.

By using data from nine consecutive cycles of NHANES with additional comprehensive information on sociodemographics, health-related factors and added sugars intake and sources, we were able to conduct a rigorous analysis of added sugars trends over an 18 year time span among U.S. adults. Furthermore, our analyses of added sugars intakes according to the health-related factors, physical activity and body weight, provide a novel contribution to the literature on time trends in added sugars intake. However, NHANES is a cross-sectional survey, with mean added sugars intakes in each cycle representing intakes of different cohorts, and so differences in intakes among cycles do not reflect changes in intakes of the same individuals. Nonetheless, changes over time in mean added sugars intake do reflect changes in intakes at the population level, and thus can be interpreted in the context of other changes over this same time period that might influence added sugars intake in the population, such as dietary recommendations and interventions. Lastly, our findings serve as a baseline against which further shifts in added sugars intakes can be assessed following the full implementation of the updated Nutrition Facts label, which displays added sugars content and Daily Value on food labels.

Any interpretation of trends over time in added sugars intake must consider other changes occurring over the same time period that might contribute to observed trends, such as changes in survey sampling, data collection, variable definitions and dietary reporting. Changes in survey sampling were accounted for with statistical adjustments, and the collection of dietary intake data and definition of added sugars were relatively stable over time, such that these factors likely had little impact on our observed trends. The lack of nationally representative data for Asian and Hispanic individuals limited our comparisons among difference race and ethnicities; however, the trends we observed for Asian and Hispanic individuals over the shorter time frame (2011–2018) can serve as a baseline. Our results are also subject to errors of misreporting as dietary intake was self-reported. Variations in energy misreporting among different age, ethnic and income groups, and among different groups defined by body weight status have been documented (32); such variations could have impacted our analyses of subsamples stratified by these factors, but this impact was minimized by expressing added sugars intake as a percentage of energy intake.

In conclusion, over the 18 year time span, from 2001 to 2018, added sugars intake declined significantly among younger adults (19–50 years) in the U.S., regardless of race and ethnicity (i.e., similar for Black and White individuals), income level, physical activity level or body weight status, and declines were mainly due to reductions in added sugars intake from sweetened beverages (primarily soft drinks and fruit drinks). These trends coincide with the evolving emphasis in the DGA on reducing added sugars intake and the increasing focus on population-level interventions aimed at reducing intakes. Yet disparities remain, with Black and low income individuals having the highest added sugars intakes, and Asian and high income individuals having the lowest. In contrast, differences in intake between younger and older adults largely disappeared after 18 years because higher intakes among younger adults fell, while lower intakes among older adults did not change. With the updated Nutrition Facts label now displaying added sugars content, there will likely be more shifts in the formulation and consumption of various food and beverage sources of added sugars, and it remains to be seen how added sugars intake trends carry forward in the future. These data could be considered baseline trends in added sugars intake from which to compare the impact of the updated Nutrition Facts label.

## Data availability statement

The data used in this study are openly available in the NHANES website: NHANES Questionnaires, Datasets, and Related Documentation https://wwwn.cdc.gov/nchs/nhanes/Default.aspx.

## Ethics statement

The NHANES study procedures are approved by an Institutional Ethics Review Board, and documented consent is obtained from NHANES participants.

## Author contributions

VF, PG, and MS designed the research. VF conducted the research and performed the statistical analysis. LD, VF, and LR analyzed the data. LD and LR wrote the manuscript. All authors reviewed and edited the manuscript drafts, and read and agreed to the published version of the manuscript.

## Funding

The authors declare that this study received funding from The Sugar Association, Inc.

## Conflict of interest

Authors LD and LR as independent consultants provide nutrition and regulatory consulting to various food manufacturers, commodity groups, and health organizations. Author VF as Vice President of Nutrition Impact, LLC conducts NHANES analyses for numerous members of the food, beverage and dietary supplement industry. Authors PG and MS are employed by The Sugar Association, Inc.

## Publisher's note

All claims expressed in this article are solely those of the authors and do not necessarily represent those of their affiliated organizations, or those of the publisher, the editors and the reviewers. Any product that may be evaluated in this article, or claim that may be made by its manufacturer, is not guaranteed or endorsed by the publisher.

## Author disclaimer

The views expressed in the manuscript are those of the authors and do not necessarily reflect the position or policy of The Sugar Association, Inc. The Sugar Association, Inc. had no restrictions regarding publication.
