# Supplementary material for: Trends in added sugars intake and sources among U.S. adults using the National Health and Nutrition Examination Survey (NHANES) 2001–2018
Source: Front Nutr. 2022 Aug 18;9:897952. doi: 10.3389/fnut.2022.897952 (PMC9434277; doi:10.3389/fnut.2022.897952)
Supplement: Supplementary file 1 [file Data_Sheet_1.docx]

**Supplemental Table 1.** Added sugars intake among adults (19+ y, *n*=44,572), 2001-2018^1^

| **Year** | **19-50 y**  **(n=23,552)** | | **51+ y**  **(n=21,020)** | | **19+ y**  **(n=44,572)** | |
| --- | --- | --- | --- | --- | --- | --- |
|  | **Mean**^2^ **(SE)**  **grams** | **P** | **Mean (SE)**  **grams** | **P** | **Mean (SE)**  **grams** | **P** |
| 2001-02 | 96.57 (2.74) |  | 59.86 (2.28) |  | 83.35 (2.12) |  |
| 2003-04 | 100.1 (2.81) | 0.3680 | 58.65 (1.51) | 0.6609 | 83.46 (1.92) | 0.9688 |
| 2005-06 | 86.37 (2.62) | 0.0079^3^ | 58.91 (2.48) | 0.7785 | 75.28 (2.31) | 0.0111 |
| 2007-08 | 86.30 (4.05) | 0.0374 | 57.47 (1.63) | 0.3957 | 74.86 (2.88) | 0.0190 |
| 2009-10 | 81.48 (1.96) | <0.0001^3^ | 58.04 (1.59) | 0.5134 | 71.82 (1.33) | <0.0001^3^ |
| 2011-12 | 83.60 (2.29) | 0.0004^3^ | 59.72 (1.98) | 0.9651 | 73.39 (1.62) | 0.0003^3^ |
| 2013-14 | 80.74 (1.96) | <0.0001^3^ | 57.43 (1.72) | 0.3967 | 70.44 (1.53) | <0.0001^3^ |
| 2015-16 | 69.97 (2.51) | <0.0001^3^ | 59.19 (1.43) | 0.8059 | 65.02 (1.69) | <0.0001^3^ |
| 2017-18 | 72.33 (2.69) | <0.0001^3^ | 64.04 (2.17) | 0.1859 | 68.47 (1.88) | <0.0001^3^ |
| **Trend** | **Beta (SE)** | **P** | **Beta (SE)** | **P** | **Beta (SE)** | **P** |
| **Linear** | -3.35 (0.35) | <0.0001^4^ | 0.36 (0.26) | 0.1730 | -2.08 (0.26) | <0.0001^4^ |

SE, standard error

^1^Source NHANES 2001-02 to 2017-18; ^2^Based on Day-1 intake data; ^3^Significantly different (*P*<0.01) from reference cycle, NHANES 2001-02; ^4^Significant (*P*<0.01) linear trend

**Supplemental Table 2.** Added sugars intake among adults (19+ y, *n=*44,572), 2001-2018^1^

| **Year** | **19-50 y**  **(n=23,552)** | | **51+ y**  **(n=21,020)** | | **19+ y**  **(n=44,572)** | |
| --- | --- | --- | --- | --- | --- | --- |
|  | **Mean**^2^ **(SE)**  **% kcal**^3^ | **P** | **Mean (SE)**  **% kcal** | **P** | **Mean (SE)**  **% kcal** | **P** |
| 2001-02 | 16.19 (0.43) |  | 12.39 (0.35) |  | 14.82 (0.36) |  |
| 2003-04 | 16.40 (0.47) | 0.7445 | 12.45 (0.27) | 0.9001 | 14.81 (0.37) | 0.9817 |
| 2005-06 | 14.39 (0.40) | 0.0025^4^ | 11.86 (0.31) | 0.2536 | 13.37 (0.29) | 0.0019^4^ |
| 2007-08 | 15.06 (0.65) | 0.1495 | 11.93 (0.26) | 0.2964 | 13.82 (0.47) | 0.0901 |
| 2009-10 | 14.15 (0.35) | 0.0003^4^ | 11.59 (0.23) | 0.0611 | 13.09 (0.23) | 0.0001^4^ |
| 2011-12 | 14.05 (0.38) | 0.0003^4^ | 11.77 (0.30) | 0.1797 | 13.08 (0.28) | 0.0002^4^ |
| 2013-14 | 14.02 (0.23) | <0.0001^4^ | 11.31 (0.34) | 0.0290 | 12.82 (0.23) | <0.0001^4^ |
| 2015-16 | 12.37 (0.36) | <0.0001^4^ | 11.72 (0.25) | 0.1237 | 12.07 (0.24) | <0.0001^4^ |
| 2017-18 | 12.70 (0.39) | <0.0001^4^ | 11.99 (0.39) | 0.4413 | 12.37 (0.33) | <0.0001^4^ |
| **Trend** | **Beta (SE)** | **P** | **Beta (SE)** | **P** | **Beta (SE)** | **P** |
| **Linear** | -0.46 (0.05) | <0.0001^5^ | -0.08 (0.04) | 0.0834 | -0.33 (0.04) | <0.0001^5^ |

SE, standard error

^1^Source NHANES 2001-02 to 2017-18; ^2^Based on Day-1 intake data; ^3^Percentage of total daily calories; ^4^Significantly different (*P*<0.01) from reference cycle, NHANES 2001-02; ^5^Significant (*P*<0.01) linear trend

**Supplemental Table 3.** Trends in added sugars intake among adults (19+ y, *n*=44,572), 2001-2018,^1^ by stratification variables

|  | **19-50 y**  **(n=23,552)** | | | | **51+ y**  **(n=21,020)** | | | | **19+ y**  **(n=44,572)** | | | |
| --- | --- | --- | --- | --- | --- | --- | --- | --- | --- | --- | --- | --- |
|  | **2001-02** | **2017-18** | **Beta**^4^ **(SE)** | **P** | **2001-02** | **2017-18** | **Beta (SE)** | **P** | **2001-02** | **2017-18** | **Beta (SE)** | **P** |
|  | **Mean**^2^ **(SE)**  **% kcal**^3^ | **Mean (SE)**  **% kcal** |  |  | **Mean (SE)**  **% kcal** | **Mean (SE)**  **% kcal** |  |  | **Mean (SE)**  **% kcal** | **Mean (SE)**  **% kcal** |  |  |
| **All Individuals** | 16.19 (0.43) | 12.70 (0.39) | -0.46 (0.05) | <0.0001^6^ | 12.39 (0.35) | 11.99 (0.39) | -0.08 (0.04) | 0.0834 | 14.82 (0.36) | 12.37 (0.33) | -0.33 (0.04) | <0.0001^6^ |
| **Race and Ethnicity** |  | | | | | | | | | | | |
| Asian^5^ | 9.68 (0.39) | 8.55 (0.39) | -0.43 (0.18) | 0.0208 | 7.36 (0.53) | 7.34 (0.40) | -0.05 (0.21) | 0.8185 | 8.86 (0.40) | 8.09 (0.25) | -0.30 (0.15) | 0.0556 |
| Black | 18.24 (1.01) | 14.99 (0.59) | -0.43 (0.10) | 0.0001^6^ | 15.72 (0.80) | 13.55 (0.66) | -0.30 (0.09) | 0.0007^6^ | 17.50 (0.70) | 14.38 (0.47) | -0.41 (0.07) | <0.0001^6^ |
| Hispanic^5^ | 13.64 (0.38) | 11.98 (0.58) | -0.55 (0.22) | 0.0126 | 10.34 (0.74) | 11.02 (0.71) | 0.16 (0.32) | 0.6122 | 12.79 (0.40) | 11.67 (0.48) | -0.40 (0.19) | 0.0452 |
| White | 16.01 (0.54) | 12.66 (0.55) | -0.45 (0.07) | <0.0001^6^ | 12.39 (0.39) | 12.21 (0.41) | -0.03 (0.05) | 0.5540 | 14.58 (0.45) | 12.43 (0.39) | -0.29 (0.05) | <0.0001^6^ |
| **Income** |  | | | | | | | | | | | |
| PIR<1.35 | 18.00 (0.68) | 14.89 (0.63) | -0.53 (0.09) | <0.0001^6^ | 12.93 (0.68) | 14.02 (1.42) | 0.03 (0.12) | 0.7869 | 16.33 (0.40) | 14.57 (0.63) | -0.36 (0.08) | <0.0001^6^ |
| 1.35≤PIR≤1.85 | 21.17 (1.25) | 12.68 (0.84) | -0.73 (0.13) | <0.0001^6^ | 12.44 (0.79) | 12.15 (0.78) | 0.06 (0.11) | 0.5456 | 18.26 (0.93) | 12.44 (0.56) | -0.43 (0.09) | <0.0001^6^ |
| PIR>1.85 | 15.06 (0.50) | 11.76 (0.41) | -0.44 (0.06) | <0.0001^6^ | 12.20 (0.37) | 11.61 (0.42) | -0.11 (0.05) | 0.0195 | 14.01 (0.43) | 11.69 (0.38) | -0.32 (0.05) | <0.0001^6^ |
| **Physical Activity** |  | | | | | | | | | | | |
| Sedentary | 18.01 (0.83) | 14.28 (0.60) | -0.51 (0.10) | <0.0001^6^ | 13.50 (0.39) | 12.18 (0.95) | -0.12 (0.08) | 0.1277 | 15.90 (0.52) | 13.03 (0.72) | -0.36 (0.07) | <0.0001^6^ |
| Moderate | 16.91 (0.62) | 13.26 (0.80) | -0.47 (0.09) | <0.0001^6^ | 11.56 (0.43) | 12.31 (0.42) | 0.01 (0.06) | 0.8936 | 14.68 (0.45) | 12.70 (0.44) | -0.28 (0.06) | <0.0001^6^ |
| Vigorous | 14.68 (0.50) | 12.06 (0.41) | -0.36 (0.06) | <0.0001^6^ | 11.43 (0.47) | 11.40 (0.55) | -0.03 (0.07) | 0.6653 | 13.96 (0.46) | 11.85 (0.36) | -0.30 (0.05) | <0.0001^6^ |
| **Body Weight Status** |  | | | | | | | | | | | |
| Normal | 15.45 (0.58) | 12.60 (0.62) | -0.48 (0.08) | <0.0001^6^ | 12.80 (0.61) | 11.87 (0.68) | -0.15 (0.08) | 0.0632 | 14.69 (0.56) | 12.30 (0.50) | -0.39 (0.07) | <0.0001^6^ |
| Overweight | 16.46 (0.78) | 11.64 (0.39) | -0.57 (0.08) | <0.0001^6^ | 12.28 (0.54) | 11.68 (0.56) | -0.12 (0.06) | 0.0617 | 14.81 (0.61) | 11.66 (0.38) | -0.39 (0.06) | <0.0001^6^ |
| Obese | 16.44 (0.73) | 13.42 (0.47) | -0.35 (0.07) | <0.0001^6^ | 12.36 (0.48) | 12.21 (0.66) | 0.01 (0.07) | 0.8796 | 14.89 (0.38) | 12.85 (0.45) | -0.23 (0.05) | <0.0001^6^ |

PIR, poverty income ratio; SE, standard error

^1^Source NHANES 2001-02 to 2017-18; ^2^Based on Day-1 intake data; ^3^Percentage of total daily calories; ^4^Represents linear trend across all cycles (means shown for first and last cycles only); ^5^First cycle is 2011-12 because data not available for 2001-02; ^6^Significant (*P*<0.01) linear trend

**Supplemental Table 4.** Trends in sources^1^ of added sugars among adults (19+ y, *n*=44,572), 2001-2018, by race and ethnicity:^2^ food group contributions as a percent of total daily added sugars intake

| **Food**  **Group**^3^ | | **Sweetened**  **Beverages** | **Sweet Bakery**  **Products** | **Sugars** | **Candy** | **Other**  **Desserts** | **Coffee**  **and Tea** | **Ready-to-Eat**  **Cereals** | **Breads, Rolls, Tortillas** |
| --- | --- | --- | --- | --- | --- | --- | --- | --- | --- |
| **All Individuals (n=44,572)** | |  | | | | | | | |
| **Mean**^4^ **(SE)**  **% TAS** | 2001-02 | 45.0 (1.1) | 12.0 (0.6) | 8.1 (0.3) | 6.1 (0.4) | 5.7 (0.3) | 4.5 (0.4) | 3.1 (0.3) | 2.4 (0.1) |
|  | 2017-18 | 32.6 (1.6) | 12.8 (0.6) | 7.6 (0.4) | 6.4 (0.5) | 5.0 (0.4) | 9.4 (1.0) | 2.8 (0.1) | 1.8 (0.1) |
|  | Beta^5^ (SE) | -1.73 (0.14) | 0.04 (0.09) | -0.05 (0.06) | -0.02 (0.06) | -0.11 (0.05) | 0.73 (0.09) | 0.001 (0.04) | -0.03 (0.04) |
|  | P | <0.0001^7^ | 0.6229 | 0.4241 | 0.7195 | 0.0802 | 0.0001^7^ | 0.9883 | 0.3806 |
| **Asian (n=2,282)** | |  | | | | | | | |
| **Mean (SE)**  **% TAS** | 2011-12^6^ | 29.87 (2.19) | 12.78 (1.79) | 8.41 (1.18) | 6.18 (0.92) | 5.99 (0.92) | 7.03 (1.20) | 3.09 (0.52) | 3.02 (0.28) |
|  | 2017-18 | 23.12 (3.98) | 14.66 (1.52) | 11.76 (1.42) | 6.64 (1.02) | 6.53 (0.95) | 10.43 (2.45) | 2.76 (0.94) | 2.80 (0.34) |
|  | Beta (SE) | -3.35 (1.36) | 0.35 (1.02) | 1.44 (0.66) | -0.20 (0.75) | 0.48 (0.67) | 1.33 (0.56) | -0.26 (0.22) | -0.02 (0.10) |
|  | P | 0.1328 | 0.7618 | 0.1603 | 0.8184 | 0.5442 | 0.1432 | 0.3534 | 0.8281 |
| **Black (n=9,633)** | |  | | | | | | | |
| **Mean (SE)**  **% TAS** | 2001-02 | 51.78 (1.42) | 9.82 (0.76) | 8.95 (1.66) | 5.92 (0.69) | 3.14 (0.57) | 4.12 (0.60) | 3.44 (0.78) |  |
|  | 2017-18 | 38.27 (1.74) | 11.41 (0.90) | 7.87 (0.65) | 5.52 (0.65) | 3.46 (0.51) | 10.41 (0.92) | 2.86 (0.34) |  |
|  | Beta (SE) | -1.78 (0.17) | 0.27 (0.15) | -0.10 (0.09) | 0.09 (0.08) | -0.12 (0.10) | 0.85 (0.14) | 0.02 (0.07) |  |
|  | P | <0.0001^7^ | 0.1075^8^ | 0.2770 | 0.2961 | 0.2692 | 0.0004^7^ | 0.7947 |  |
| **Hispanic (n=4,734)** | |  | | | | | | | |
| **Mean (SE)**  **% TAS** | 2011-12^6^ | 45.94 (3.02) | 14.23 (1.86) | 7.97 (0.81) | 3.50 (0.65) | 3.63 (0.48) | 6.60 (1.06) | 3.16 (0.32) | 2.16 (0.21) |
|  | 2017-18 | 43.23 (2.32) | 12.12 (1.11) | 7.24 (0.63) | 5.11 (0.77) | 4.26 (0.66) | 6.51 (0.85) | 2.10 (0.33) | 1.84 (0.23) |
|  | Beta (SE) | -1.68 (1.04) | -0.06 (0.66) | -0.24 (0.05) | 0.12 (0.53) | 0.24 (0.06) | 0.08 (0.62) | -0.31 (0.16) | -0.18 (0.09) |
|  | P | 0.2484 | 0.9321 | 0.0421 | 0.8442 | 0.0557 | 0.9079 | 0.1902 | 0.1872 |
| **White (n=19,764)** | |  | | | | | | | |
| **Mean (SE)**  **% TAS** | 2001-02 | 41.62 (1.67) | 12.95 (0.72) | 8.02 (0.31) | 6.04 (0.49) | 6.61 (0.42) | 4.85 (0.49) | 3.30 (0.38) | 2.67 (0.13) |
|  | 2017-18 | 29.46 (1.87) | 12.88 (0.98) | 7.03 (0.54) | 7.13 (0.87) | 5.45 (0.50) | 9.38 (1.35) | 2.94 (0.21) | 1.82 (0.18) |
|  | Beta (SE) | -1.75 (0.18) | -0.07 (0.11) | -0.10 (0.07) | 0.04 (0.07) | -0.11 (0.05) | 0.68 (0.09) | 0.01 (0.04) | -0.02 (0.04) |
|  | P | <0.0001^7^ | 0.5369 | 0.2340 | 0.6435 | 0.0513 | 0.0001^7^ | 0.7607 | 0.5739 |

SE, standard error; TAS, total added sugars

^1^Contributing at least 2% to TAS among all individuals (2-18 y) in first cycle, NHANES 2001-02, with empty cells representing a food group contributing less than 2%; ^2^Source NHANES 2001-02 to 2017-18; ^3^2017-18 What We Eat in America food groups; ^4^Based on Day-1 intake data; ^5^Represents linear trend across all cycles (means shown for first and last cycles only); ^6^First cycle is 2011-12 because data not available for 2001-02; ^7^Significant (*P*<0.01) linear trend; ^8^Significant quadratic trend, Beta_1_=1.58 (0.24), *P*=0.0005 and Beta_2_=-0.13 (0.02), *P*=0.0012

**Supplemental Table 5.** Trends in sources^1^ of added sugars among adults (19+ y, *n*=44,572), 2001-2018, by income:^2^ food group contributions as a percent of total daily added sugars intake

| **Food**  **Group**^3^ | | **Sweetened**  **Beverages** | **Sweet Bakery**  **Products** | **Sugars** | **Candy** | **Other**  **Desserts** | **Coffee**  **and Tea** | **Ready-to-Eat**  **Cereals** | **Breads, Rolls, Tortillas** |
| --- | --- | --- | --- | --- | --- | --- | --- | --- | --- |
| **All Individuals (n=44,572)** | |  | | | | | | | |
| **Mean**^4^ **(SE)**  **% TAS** | 2001-02 | 45.0 (1.1) | 12.0 (0.6) | 8.1 (0.3) | 6.1 (0.4) | 5.7 (0.3) | 4.5 (0.4) | 3.1 (0.3) | 2.4 (0.1) |
|  | 2017-18 | 32.6 (1.6) | 12.8 (0.6) | 7.6 (0.4) | 6.4 (0.5) | 5.0 (0.4) | 9.4 (1.0) | 2.8 (0.1) | 1.8 (0.1) |
|  | Beta^5^ (SE) | -1.73 (0.14) | 0.04 (0.09) | -0.05 (0.06) | -0.02 (0.06) | -0.11 (0.05) | 0.73 (0.09) | 0.001 (0.04) | -0.03 (0.04) |
|  | P | <0.0001^6^ | 0.6229 | 0.4241 | 0.7195 | 0.0802 | 0.0001^6^ | 0.9883 | 0.3806 |
| **PIR<1.35 (n=13,355)** | |  | | | | | | | |
| **Mean (SE)**  **% TAS** | 2001-02 | 53.73 (2.13) | 9.50 (1.06) | 8.19 (0.74) | 4.72 (0.35) | 3.53 (0.46) | 5.39 (0.97) | 2.45 (0.30) | 2.05 (0.15) |
|  | 2017-18 | 43.33 (2.61) | 10.15 (0.98) | 8.05 (1.08) | 4.67 (0.66) | 3.17 (0.46) | 10.47 (0.92) | 2.75 (0.38) | 1.35 (0.15) |
|  | Beta (SE) | -1.89 (0.29) | 0.14 (0.12) | -0.02 (0.08) | -0.08 (0.07) | -0.07 (0.08) | 0.88 (0.16) | 0.06 (0.05) | -0.04 (0.04) |
|  | P | 0.0003^6^ | 0.2750 | 0.7970 | 0.3237 | 0.3983 | 0.0008^6^ | 0.2724 | 0.3469 |
| **1.35≤PIR≤1.85 (n=5,046)** | |  | | | | | | | |
| **Mean (SE)**  **% TAS** | 2001-02 | 55.21 (3.37) | 9.69 (0.91) | 6.75 (1.11) | 4.95 (0.98) | 5.17 (1.04) | 3.24 (0.65) | 2.22 (0.38) |  |
|  | 2017-18 | 37.80 (3.10) | 12.54 (1.13) | 6.58 (1.01) | 4.55 (0.70) | 4.96 (0.79) | 9.55 (1.96) | 4.05 (0.52) |  |
|  | Beta (SE) | -1.92 (0.29) | 0.20 (0.22) | 0.00 (0.06) | -0.09 (0.08) | -0.02 (0.10) | 1.17 (0.26) | 0.13 (0.06) |  |
|  | P | 0.0003^6^ | 0.3950 | 0.9874 | 0.3170 | 0.8475 | 0.0028^6^ | 0.0576 |  |
| **PIR>1.85 (n=22,609)** | |  | | | | | | | |
| **Mean (SE)**  **% TAS** | 2001-02 | 40.70 (1.49) | 13.19 (0.68) | 8.08 (0.39) | 6.74 (0.54) | 6.55 (0.46) | 4.53 (0.43) | 3.45 (0.42) | 2.67 (0.11) |
|  | 2017-18 | 26.97 (1.62) | 14.17 (0.98) | 7.39 (0.53) | 7.41 (0.96) | 5.77 (0.56) | 9.07 (1.25) | 2.58 (0.21) | 1.83 (0.14)^G^ |
|  | Beta (SE) | -1.89 (0.16) | 0.02 (0.08) | -0.04 (0.07) | 0.05 (0.09) | -0.06 (0.07) | 0.66 (0.08) | -0.03 (0.05) | -0.05 (0.04) |
|  | P | <0.0001^6^ | 0.8307 | 0.5681 | 0.6474 | 0.3932 | 0.0001^6^ | 0.6557 | 0.2772 |

PIR, poverty income ratio; SE, standard error; TAS, total added sugars

^1^Contributing at least 2% to TAS among all individuals (2-18 y) in first cycle, NHANES 2001-02, with empty cells representing a food group contributing less than 2%; ^2^Source NHANES 2001-02 to 2017-18; ^3^2017-18 What We Eat in America food groups; ^4^Based on Day-1 intake data; ^5^Represents linear trend across all cycles (means shown for first and last cycles only); ^6^Significant (*P*<0.01) linear trend

**Supplemental Table 6.** Trends in sources^1^ of added sugars among adults (19+ y, *n*=44,572), 2001-2018, by physical activity:^2^ food group contributions as a percent of total daily added sugars intake

| **Food**  **Group**^3^ | | **Sweetened Beverages** | **Sweet Bakery Products** | **Sugars** | **Candy** | **Other**  **Desserts** | **Coffee**  **and Tea** | **Ready-to-Eat Cereals** | **Breads, Rolls, Tortillas** |
| --- | --- | --- | --- | --- | --- | --- | --- | --- | --- |
| **All Individuals (n=44,572)** | |  | | | | | | | |
| **Mean**^4^ **(SE)**  **% TAS** | 2001-02 | 45.0 (1.1) | 12.0 (0.6) | 8.1 (0.3) | 6.1 (0.4) | 5.7 (0.3) | 4.5 (0.4) | 3.1 (0.3) | 2.4 (0.1) |
|  | 2017-18 | 32.6 (1.6) | 12.8 (0.6) | 7.6 (0.4) | 6.4 (0.5) | 5.0 (0.4) | 9.4 (1.0) | 2.8 (0.1) | 1.8 (0.1) |
|  | Beta^5^ (SE) | -1.73 (0.14) | 0.04 (0.09) | -0.05 (0.06) | -0.02 (0.06) | -0.11 (0.05) | 0.73 (0.09) | 0.001 (0.04) | -0.03 (0.04) |
|  | P | <0.0001^6^ | 0.6229 | 0.4241 | 0.7195 | 0.0802 | 0.0001^6^ | 0.9883 | 0.3806 |
| **Sedentary (n=13,611)** | |  | | | | | | | |
| **Mean (SE)**  **% TAS** | 2001-02 | 47.5 (1.5) | 12.4 (0.8) | 8.4 (0.4) | 5.1 (0.3) | 5.2 (0.5) | 5.2 (0.6) | 2.6 (0.4) | 2.3 (0.1) |
|  | 2017-18 | 32.3 (2.1) | 13.9 (1.6) | 7.8 (1.3) | 6.2 (1.0) | 4.5 (0.7) | 10.5 (2.0) | 2.5 (0.4) | 1.8 (0.2) |
|  | Beta (SE) | -2.24 (0.29) | 0.18 (0.15) | -0.001 (0.10) | 0.04 (0.06) | -0.03 (0.10) | 0.91 (0.26) | 0.05 (0.04) | -0.04 (0.03) |
|  | P | 0.0001^6^ | 0.2752 | 0.9887 | 0.5754 | 0.7643 | 0.0102 | 0.2312 | 0.2254^7^ |
| **Moderate (n=15,418)** | |  | | | | | | | |
| **Mean (SE)**  **% TAS** | 2001-02 | 45.2 (2.1) | 12.2 (1.2) | 8.2 (0.5) | 6.0 (0.8) | 5.4 (0.4) | 4.6 (0.8) | 3.1 (0.4) | 2.5 (0.2) |
|  | 2017-18 | 32.2 (1.3) | 13.3 (0.8) | 7.5 (0.7) | 7.5 (1.4) | 5.9 (0.5) | 7.7 (1.1) | 2.7 (0.3) | 1.7 (0.2) |
|  | Beta (SE) | -1.49 (0.13) | 0.01 (0.14) | -0.11 (0.06) | 0.01 (0.08) | 0.001 (0.06) | 0.49 (0.15) | 0.001 (0.05) | -0.06 (0.04) |
|  | P | <0.0001^6^ | 0.9195 | 0.1391 | 0.9289 | 0.9893 | 0.0118 | 0.9871 | 0.2259^8^ |
| **Vigorous (n=15,532)** | |  | | | | | | | |
| **Mean (SE)**  **% TAS** | 2001-02 | 42.8 (1.5) | 11.5 (1.3) | 7.7 (0.5) | 7.1 (0.9) | 6.4 (0.5) | 3.9 (0.5) | 3.5 (0.4) | 2.6 (0.1) |
|  | 2017-18 | 32.9 (2.4) | 11.9 (1.0) | 7.7 (0.6) | 5.6 (0.8) | 4.6 (0.5) | 10.2 (1.0) | 2.9 (0.2) | 1.8 (0.1) |
|  | Beta (SE) | -1.36 (0.22) | -0.02 (0.17) | 0.03 (0.08) | -0.21 (0.13) | -0.22 (0.05) | 0.87 (0.09) | -0.05 (0.04) | -0.05 (0.04) |
|  | P | 0.0005^6^ | 0.9024 | 0.7633 | 0.1524 | 0.0040^6^ | <0.0001^6^ | 0.2533 | 0.2004 |

SE, standard error; TAS, total added sugars

^1^Contributing at least 2% to TAS among all individuals (19+ y) in first cycle, NHANES 2001-02; ^2^Source NHANES 2001-02 to

2017-18; ^3^2017-18 What We Eat in America food groups; ^4^Based on Day-1 intake data; ^5^Represents linear trend across all cycles (means shown for first and last cycles only); ^6^Significant (*P*<0.01) linear trend; ^7^Significant quadratic trend, Beta_1_=0.28 (0.07), *P*=0.0059 and Beta_2_=-0.03 (0.01), *P*=0.0030; ^8^Significant quadratic trend, Beta_1_=0.42 (0.08), *P*=0.0019 and Beta_2_=-0.05 (0.01), *P*=0.0009

**Supplemental Table 7.** Trends in sources^1^ of added sugars among adults (19+ y, *n*=44,572), 2001-2018, by body weight status:^2^ food group contributions as a percent of total daily added sugars intake

| **Food**  **Group**^3^ | | **Sweetened Beverages** | **Sweet Bakery Products** | **Sugars** | **Candy** | **Other**  **Desserts** | **Coffee**  **and Tea** | **Ready-to-Eat Cereals** | **Breads, Rolls, Tortillas** |
| --- | --- | --- | --- | --- | --- | --- | --- | --- | --- |
| **All Individuals (n=44,572)** | |  | | | | | | | |
| **Mean**^4^ **(SE)**  **% TAS** | 2001-02 | 45.0 (1.1) | 12.0 (0.6) | 8.1 (0.3) | 6.1 (0.4) | 5.7 (0.3) | 4.5 (0.4) | 3.1 (0.3) | 2.4 (0.1) |
|  | 2017-18 | 32.6 (1.6) | 12.8 (0.6) | 7.6 (0.4) | 6.4 (0.5) | 5.0 (0.4) | 9.4 (1.0) | 2.8 (0.1) | 1.8 (0.1) |
|  | Beta^5^ (SE) | -1.73 (0.14) | 0.04 (0.09) | -0.05 (0.06) | -0.02 (0.06) | -0.11 (0.05) | 0.73 (0.09) | 0.001 (0.04) | -0.03 (0.04) |
|  | P | <0.0001^6^ | 0.6229 | 0.4241 | 0.7195 | 0.0802 | 0.0001^6^ | 0.9883 | 0.3806 |
| **Normal Weight (n=12,446)** | |  | | | | | | | |
| **Mean (SE)**  **% TAS** | 2001-02 | 42.3 (1.8) | 12.9 (1.3) | 9.3 (0.5) | 6.8 (0.6) | 5.5 (0.7) | 3.5 (0.6) | 3.6 (0.4) | 2.5 (0.1) |
|  | 2017-18 | 32.8 (3.8) | 12.7 (1.6) | 7.9 (1.0) | 9.3 (1.8) | 5.6 (0.9) | 7.6 (1.1) | 3.5 (0.3) | 1.5 (0.2) |
|  | Beta (SE) | -1.77 (0.32) | -0.05 (0.06) | -0.17 (0.10) | -0.21 (0.13) | -0.18 (0.11) | 0.76 (0.17) | 0.05 (0.04) | -0.03 (0.04) |
|  | P | 0.0008^6^ | 0.4338 | 0.1366 | 0.1528 | 0.1481 | 0.0025^6^ | 0.3245 | 0.4627 |
| **Overweight (n=14,611)** | |  | | | | | | | |
| **Mean (SE)**  **% TAS** | 2001-02 | 43.8 (1.4) | 12.5 (0.5) | 8.5 (0.5) | 5.7 (0.7) | 5.9 (0.5) | 5.1 (0.5) | 3.1 (0.4) | 2.4 (0.2) |
|  | 2017-18 | 25.4 (1.6) | 15.9 (1.5) | 9.5 (0.5) | 5.0 (0.8) | 4.5 (0.6) | 11.1 (2.0) | 2.7 (0.3) | 2.0 (0.2) |
|  | Beta (SE) | -2.24 (0.24) | 0.22 (0.12) | 0.07 (0.14) | -0.004 (0.08) | -0.10 (0.07) | 0.52 (0.16) | 0.02 (0.05) | -0.02 (0.04) |
|  | P | <0.0001^6^ | 0.1157 | 0.6371 | 0.9658 | 0.1991 | 0.0164 | 0.6653 | 0.5939 |
| **Obese (n=16,041)** | |  | | | | | | | |
| **Mean (SE)**  **% TAS** | 2001-02 | 48.6 (2.5) | 10.8 (0.9) | 6.4 (0.5) | 5.7 (0.6) | 5.8 (0.6) | 5.6 (1.1) | 2.5 (0.4) | 2.5 (0.1) |
|  | 2017-18 | 35.9 (1.9) | 11.2 (0.7) | 6.4 (0.7) | 5.6 (0.6) | 5.1 (0.5) | 9.7 (1.0) | 2.3 (0.2) | 1.8 (0.1) |
|  | Beta (SE) | -1.49 (0.28) | -0.03 (0.14) | 0.10 (0.07) | -0.03 (0.05) | -0.21 (0.08) | 0.71 (0.12) | -0.01 (0.05) | -0.08 (0.04) |
|  | P | 0.0012^6^ | 0.8267 | 0.1995 | 0.6387 | 0.0377 | 0.0005^6^ | 0.8106 | 0.0566 |

SE, standard error; TAS, total added sugars

^1^Contributing at least 2% to TAS among all individuals (19+ y) in first cycle, NHANES 2001-02; ^2^Source NHANES 2001-02 to

2017-18; ^3^2017-18 What We Eat in America food groups; ^4^Based on Day-1 intake data; ^5^Represents linear trend across all cycles (means shown for first and last cycles only); ^6^Significant (*P*<0.01) linear trend
